# Generation and Efficacy of Two Chimeric Viruses Derived from GPE^−^ Vaccine Strain as Classical Swine Fever Vaccine Candidates

**DOI:** 10.3390/v15071587

**Published:** 2023-07-20

**Authors:** Loc Tan Huynh, Norikazu Isoda, Lim Yik Hew, Saho Ogino, Yume Mimura, Maya Kobayashi, Taksoo Kim, Tatsuya Nishi, Katsuhiko Fukai, Takahiro Hiono, Yoshihiro Sakoda

**Affiliations:** 1 Laboratory of Microbiology, Department of Disease Control, Faculty of Veterinary Medicine, Hokkaido University, Sapporo 060-0818, Hokkaido, Japan; huynhtanloc@vetmed.hokudai.ac.jp (L.T.H.); nisoda@vetmed.hokudai.ac.jp (N.I.); ylhew@vetmed.hokudai.ac.jp (L.Y.H.); microbio-data@vetmed.hokudai.ac.jp (S.O.); yume1101@eis.hokudai.ac.jp (Y.M.); jya1225kuchu@eis.hokudai.ac.jp (M.K.); taksoo.kim@wisc.edu (T.K.); hiono@vetmed.hokudai.ac.jp (T.H.); 2 Faculty of Veterinary Medicine, College of Agriculture, Can Tho University, Can Tho 900000, Vietnam; 3 One Health Research Center, Hokkaido University, Sapporo 060-0818, Hokkaido, Japan; 4 International Collaboration Unit, International Institute for Zoonosis Control, Hokkaido University, Sapporo 001-0020, Hokkaido, Japan; 5 Institute for Vaccine Research and Development (HU-IVReD), Hokkaido University, Sapporo 001-0021, Hokkaido, Japan; 6 Kodaira Research Station, National Institute of Animal Health, National Agriculture and Food Research Organization, Kodaira 187-0022, Tokyo, Japan; ultra1124@affrc.go.jp (T.N.); fukai@affrc.go.jp (K.F.)

**Keywords:** chimeric virus, vaccine efficacy, classical swine fever virus, pestivirus, GPE^−^

## Abstract

A previous study proved that vGPE^−^ mainly maintains the properties of classical swine fever (CSF) virus, which is comparable to the GPE^−^ vaccine seed and is a potentially valuable backbone for developing a CSF marker vaccine. Chimeric viruses were constructed based on an infectious cDNA clone derived from the live attenuated GPE^−^ vaccine strain as novel CSF vaccine candidates that potentially meet the concept of differentiating infected from vaccinated animals (DIVA) by substituting the glycoprotein E^rns^ of the GPE^−^ vaccine strain with the corresponding region of non-CSF pestiviruses, either pronghorn antelope pestivirus (PAPeV) or Phocoena pestivirus (PhoPeV). High viral growth and genetic stability after serial passages of the chimeric viruses, namely vGPE^−^/PAPeV E^rns^ and vGPE^−^/PhoPeV E^rns^, were confirmed in vitro. In vivo investigation revealed that two chimeric viruses had comparable immunogenicity and safety profiles to the vGPE^−^ vaccine strain. Vaccination at a dose of 10^4.0^ TCID_50_ with either vGPE^−^/PAPeV E^rns^ or vGPE^−^/PhoPeV E^rns^ conferred complete protection for pigs against the CSF virus challenge in the early stage of immunization. In conclusion, the characteristics of vGPE^−^/PAPeV E^rns^ and vGPE^−^/PhoPeV E^rns^ affirmed their properties, as the vGPE^−^ vaccine strain, positioning them as ideal candidates for future development of a CSF marker vaccine.

## 1. Introduction

Classical swine fever (CSF) is a contagious viral disease severely affecting domestic pigs and wild boars with high mortality. It poses a significant threat to pig populations worldwide, leading to severe economic losses, trade restrictions, and animal welfare concerns [1,2]. The disease is caused by the CSF virus (CSFV), a member of the Pestivirus genus within the Flaviviridae family. The phylogeny of CSFV is closely related to bovine viral diarrhea virus (BVDV) and border disease virus (BDV) as well as several additional species that are found as atypical pestiviruses [3,4]. The CSFV genome is a single-stranded positive-sense RNA of approximately 12.3 kb in length with an open reading frame (ORF) flanked by two untranslated regions (UTRs), 5′-UTR and 3′-UTR. The ORF encodes a polyprotein of approximately 4000 amino acids (aa), which is cleaved by cellular and viral proteases cotranslationally and post-translationally into twelve proteins, including four structural proteins (C, E^rns^, E1, and E2) and eight non-structural proteins (N^pro^, p7, NS2, NS3, NS4A, NS4B, NS5A, and NS5B) [5,6].

In Japan, the principal approach to control CSF is the vaccination of domestic pigs with a live attenuated vaccine strain GPE^−^, which has been extensively used for decades, resulting in CSF-free status in 2007 by the World Organisation for Animal Health. However, the outbreak of CSF in 2018 in Japan led to the challenge of eradicating CSF after more than two decades of successful eradication programs [7,8]. Extensive vaccination using live attenuated vaccines has been implemented and widely applied in several countries as a mandatory CSF control program, along with other biosafety approaches [9]. Although the advantage of the above vaccines is to provide complete protection against CSF, a drawback of vaccines is the lack of an immunological marker for differentiating infected from vaccinated animals (DIVA), making proof of CSF eradication difficult and inhibiting international pig trade [10,11]. In this scenario, the ideal vaccine can be engineered by combining live attenuated vaccines with marker properties [10,12].

Several strategies for developing CSF marker vaccines have been broadly investigated, including modified live attenuated vaccine (MLV), viral vector vaccine, and subunit vaccine [13]. In this regard, the MLV based on the chimeric pestivirus concept was considered a promising genetic construction to enable the DIVA system by combining the efficacy of the live attenuated vaccine with a serological marker [10]. Several CSF marker vaccine candidates have recently been generated based on the phylogeny of closely related pestiviruses to CSFV [14,15,16,17]. On this subject, the chimeric vaccine harboring the E2 sequence of the CSFV Alfort/187 strain based on the backbone of the BVDV CP7 strain, namely CP_E2alf [16], and the mutant chimeric virus based on the backbone of the live attenuated CSFV LOM strain with the substitution of the complete BVDV E^rns^ sequence, namely Flc-LOM-BE^rns^ [17], have been licensed and approved as live marker vaccines in Europe and Korea, respectively [17,18]. Nevertheless, the cross-reactivity due to the genetically close relationship among pestiviruses could not satisfy the DIVA strategy [19,20]. Therefore, improvements in the chimeric virus’s construction should be considered to overcome this drawback.

Considering the developments of CSF marker vaccine candidates and addressing the issue of chimeric pestiviruses closely related to CSFV, a scheme of chimera construction based on substituting viral glycoprotein E^rns^ has been employed to design chimeric pestiviruses that are genetically and antigenically distant from CSFV [21]. Consequently, three chimeric viruses, namely “Ra”, “Pro”, and “RaPro”, have been generated by substituting the E^rns^ sequence of the CSFV Alfort-Tübingen strain with those of Norway rat pestivirus (NRPV) and pronghorn antelope pestivirus (PAPeV) or a combination of both, respectively [21]. However, several concerns about these chimeric viruses could arise with the use of the virulent CSFV strain as a backbone. As a result, the viral genome was still detected in the organ tissues of several vaccinated pigs, indicating that additional attenuation is required to obtain a safe vaccine [21]. In this case, a combination of live attenuated vaccine strains as the backbone with a gene marker genetically and antigenically distant from CSFV would be novel for chimeric virus construction.

Previous studies have established the reverse genetic system for the GPE^−^ vaccine strain, resulting in the generation of the vGPE^−^ strain consisting of 10 aa substitutions compared to the GPE^−^ vaccine seed [22,23,24]. In addition, an advanced study was conducted to investigate the effects of these 10 aa substitutions, indicating that the vGPE^−^ primarily preserves properties comparable to the GPE^−^ vaccine strain and is potentially valuable for developing a CSF marker vaccine [25]. In this regard, constructing chimeric viruses with a marker property based on the vGPE^−^ strain backbone would aid in the development of novel CSF vaccine candidates with the potential DIVA property as well as advance the ongoing CSF eradication campaign in Japan.

This study was part of a series of research projects aimed at engineering the GPE^−^ vaccine strain into a chimeric virus vaccine candidate possessing a potential immunological marker that may be used in the future to develop a novel CSF marker vaccine in the future. By replacing the complete viral glycoprotein E^rns^ of the vGPE^−^ strain with those of non-CSFV pestiviruses, either PAPeV or Phocoena pestivirus (PhoPeV), two chimeric viruses, namely vGPE^−^/PAPeV E^rns^ and vGPE^−^/PhoPeV E^rns^, were generated. The viral growth and genetic stability of the two chimeric viruses were evaluated in vitro. In addition, the optimal dose of the chimeric viruses to induce protection was also determined in pig experiments. The early-onset protection of these chimeric viruses was further evaluated in challenge studies with a moderately virulent CSFV strain 7 days after of vaccination.

## 2. Materials and Methods

### 2.1. Cells and Viruses

The swine kidney-derived cell line (SK-L cells) [26] was cultured in Eagle’s minimum essential medium (EMEM) (Nissui Pharmaceutical, Tokyo, Japan) supplemented with 0.295% tryptose phosphate broth (TPB) (Becton Dickinson, Franklin Lakes, NJ, USA), 10 mM N,N-bis-(2-hydroxyethyl)-2-aminoethanesulfonic acid (BES) (Sigma-Aldrich, St. Louis, MO, USA), sodium bicarbonate (Nacalai Tesque, Kyoto, Japan), and 10% horse serum (HS; Thermo Fisher Scientific, Waltham, MA, USA). SK-L cells were used for viral production, titration, and serological tests. The cell line was incubated at 37 °C in 5% CO_2_.

A recombinant clone of CSFV live attenuated vaccine, vGPE^−^, was derived from pGPE^−^ [22], and a plasmid containing its full-length cDNA was used. A CSFV vALD-A76 was also derived from the full-length cDNA clone of a virulent strain, ALD-A76, which was developed in a previous study [27].

### 2.2. Construction of Chimeric Pestiviruses

The chimeric viruses were constructed by substituting the complete E^rns^ sequence of vGPE^−^ with that of non-CSFV pestiviruses, either PAPeV (GeneBank accession number NC02418.2) or PhoPeV isolate NS170386 (GeneBank accession number MK910229.1), which are genetically distant from CSFV. These two chimeric viruses were constructed based on the backbone of an infectious cDNA clone pGPE^−^ [22] of a CSFV live attenuated vaccine GPE^−^ using in-fusion cloning. PAPeV E^rns^ or PhoPeV E^rns^ insert fragments (each cDNA gene was synthesized by Fasmac Co., Ltd., Kanagawa, Japan) and pGPE^−^ vector fragment were amplified by specific in-fusion polymerase chain reaction (PCR) primers using the KOD FX Neo (TOYOBO, Osaka, Japan) and the In-Fusion HD Cloning Kit (TaKaRa Bio, Shiga, Japan).

As the insertion of the PhoPeV E^rns^ sequence is toxic to bacteria, the plasmid of pGPE^−^/PhoPeV E^rns^ could not be stably replicated in *Escherichia coli*. Therefore, the full length of the vGPE^−^/PhoPeV E^rns^ genome was constructed using the OriCiro^®^ Cell-Free Cloning System [28] (Oriciro Genomics, Inc., Tokyo, Japan). The circular DNA of GPE^−^/PhoPeV E^rns^ was constructed as described in the OriCiro^®^ Cell-Free Cloning System Manual version 4.1, released in January 2021. Briefly, the circular DNA of GPE^−^/PhoPeV E^rns^ was constructed by applying the system consisting of two kits. In the first step, the OriCiro Assembly Kit allows the seamless assembly of six overlapping DNA fragments (forty nucleotides overlap), of which six fragments were generated by PCR using the KOD FX Neo. In the next step, the assembly product was directly added to the OriCiro Amp Kit for selective GPE^−^/PhoPeV E^rns^ circular DNA amplification. The template was amplified by PCR (AccuPrime™ Taq DNA Polymerase System, Thermo Fisher Scientific) using a primer containing the T7 promoter sequence (TAATACGACTCACTATAG). The recovered cDNA with the target sequence was isolated using electrophoresis, followed by gel cutting and recovery. The full-length linear cDNA was confirmed by Sanger sequencing and used as a transcriptional template for virus rescue.

### 2.3. Virus Rescue

The cDNA-derived chimeric viruses were rescued as described previously [22,29]. Briefly, the plasmid pGPE^−^/PAPeV E^rns^ was linearized at the SrfI site at the 3′-end of the viral genomic cDNA sequence and purified. The linearized product of pGPE^−^/PAPeV E^rns^ and the full-length chimeric cDNA template of pGPE^−^/PhoPeV E^rns^ were used for run-off transcription with the MEGAscript T7 kit (Thermo Fisher Scientific). After DNase I digestion and purification on MicroSpin S-400 HR columns (Cytiva, Global Life Sciences Solutions Operations UK Ltd., Buckinghamshire, UK), RNA was quantified using NanoDrop One (Thermo Fisher Scientific) and transfected into SK-L cells by electroporation using a Gene Pulser Xcell Electroporation System (Bio-Rad, Hercules, CA, USA), set at 200 V and 500 µF, followed by incubation at 37 °C for 72 h. Immunoperoxidase (IPX) staining was performed to confirm virus recovery using an anti-NS3 monoclonal antibody (mAb) 46/1, as described previously [30].

### 2.4. Genetic Stability Assessment

The genetic stability of each virus was evaluated during blind passages [31]. The parental and chimeric viruses were passaged on SK-L cells for five rounds, and 100 μL of the culture supernatants was used to infect naïve cells. At 72 h after inoculation, the culture supernatants were collected for viral titration and subjected to the next passage. The virus titers were determined in triplicate using SK-L cells. The cell culture supernatant of each passage was utilized for sequencing to confirm the genetic stability.

### 2.5. Sequencing

As described previously [22], the full-length cDNA clones of each chimeric virus and the entire genomes of rescued viruses were verified. Briefly, nucleotide sequencing of cDNA clones and PCR fragments from viral RNA was performed using the BigDye Terminator version 3.1 Cycle Sequencing Kit (Thermo Fisher Scientific, Waltham, MA, USA) and an ABI 3500 Genetic Analyzer (Thermo Fisher Scientific). Sequencing data were then analyzed using the GENETYX^®^ Network Edition version 15.0.1 software (GENETYX, Tokyo, Japan).

### 2.6. Virus Titration

For virus titration, a serial 10-fold diluted viral stock was added to SK-L cells in 96-well plates. Cells were air-dried and heat-fixed at 80 °C after 96 h of incubation at 37 °C in 5% CO_2_, followed by staining with anti-NS3 mAb 46/1 as the primary antibody for IPX staining [30]. The viral titers were calculated using the method of Reed and Muench and expressed as 50% tissue culture infective dose per milliliter (TCID_50_/mL) [32].

### 2.7. Animal Use

Prophylactic vaccination in domestic pigs in Japan has been carried out since October 2019. The vaccination area has been expanded to most regions, excluding the islands of Hokkaido and Kyushu. In this study, pigs were purchased from a CSFV-free farm in Hokkaido (Yamanaka Chikusan, Hokkaido, Japan) that had been proven to be free of antibodies against CSFV.

### 2.8. Animal Experiments

To assess the pathogenicity of vGPE^−^/PAPeV E^rns^ and vGPE^−^/PhoPeV E^rns^, five 2-week-old crossbred landrace × Duroc × Yorkshire SPF pigs (Yamanaka Chikusan) per independent experiment were inoculated intramuscularly with 10^7.0^ TCID_50_ of each virus. All pigs were monitored daily for clinical scores according to a scoring system for 14 days [33]. Blood and serum samples were collected in tubes containing ethylenediaminetetraacetic acid (EDTA) (Venoject II VP-NA050K; Terumo, Tokyo, Japan) and blood coagulation factor (Venoject II VP-P075K; Terumo), respectively, at 0, 3, 5, 7, 9, 11, and 14 days post-inoculation (dpi). The total numbers of leukocytes and thrombocytes were counted using a pocH-100iV Diff apparatus (Sysmex, Hyogo, Japan). All surviving pigs were euthanized at 14 dpi. The levels of CSFV-specific neutralizing antibodies in pigs at 0 and 14 dpi were evaluated via a serological test. Organ samples were collected aseptically, including tonsils, brains, spleens, adrenal glands, kidneys, mesenteric lymph nodes, and colons. For virus titration, 10% organ homogenates were prepared in 10% HS in EMEM and centrifuged at 3000 rpm for 5 min. The virus titers were then measured and displayed as TCID_50_/mL (blood) or TCID_50_/g (tissue).

To evaluate the optimal infectious dose and immune responses of vGPE^−^, vGPE^−^/PAPeV E^rns^, and vGPE^−^/PhoPeV E^rns^, nine 2-week-old crossbred landrace × Duroc × Yorkshire SPF pigs per independent experiment were inoculated intramuscularly with different doses of TCID_50_. Three subgroups of three pigs were inoculated with 10^3.0^, 10^4.0^, and 10^5.0^ TCID_50_ of the virus, respectively. All pigs were monitored daily for clinical scores according to a scoring system for 21 days [33]. Blood and serum samples were collected in tubes containing EDTA (Venoject II VP-NA050K; Terumo) and blood coagulation factor (Venoject II VP-P075K; Terumo), respectively, at 0, 7, 14, and 21 dpi. The total numbers of leukocytes and thrombocytes were counted by a pocH-100iV Diff apparatus (Sysmex, Hyogo, Japan). All pigs were euthanized at 21 dpi. The levels of CSFV-specific neutralizing antibodies of pigs at 0, 7, 14, and 21 dpi were evaluated by a serological test. Virus yield was titrated and expressed as TCID_50_/mL in blood samples.

The vaccine efficacy of each chimeric virus was evaluated. Due to the limitations of the present experimental conditions, vaccine efficacy studies were undertaken independently via two animal trials. In the first trial, nine 2-week-old crossbred landrace × Duroc × Yorkshire SPF pigs were randomly divided into three groups: the vGPE^−^ vaccination group (*n* = 3), vGPE^−^/PAPeV E^rns^ vaccination group (*n* = 3), and control group (*n* = 3). In the second trial, six SPF pigs were divided into the vGPE^−^/PhoPeV E^rns^ vaccination group (*n* = 3) and the control group (*n* = 3). These two trials were conducted under the same conditions: pigs in each vaccination group were intramuscularly injected with 10^4.0^ TCID_50_ of vGPE^−^ or each chimeric virus on day 0, and pigs in each control group were intramuscularly injected with 1 × phosphate-buffered saline (PBS). The serum samples were collected at 0 and 7 days post-vaccination (dpv) to detect CSFV-specific neutralizing antibodies. At 7 dpv, pigs were intranasally inoculated with 10^6.0^ TCID_50_ of the moderately virulent CSFV vALD-A76 strain and monitored daily for body temperature and clinical scores [33]. All surviving pigs were euthanized at 14 days post-challenge (dpc). Organ samples were collected aseptically, including tonsils, brains, spleens, adrenal glands, kidneys, mesenteric lymph nodes, and colons. For virus titration, the 10% organ homogenates were prepared in 10% HS in EMEM and centrifuged at 3000 rpm for 5 min. The virus titers were then measured and displayed as TCID_50_/mL (blood) or TCID_50_/g (tissue).

### 2.9. Serum Neutralization Test (SNT)

The SNT was conducted using a luciferase-based assay, as previously described [34]. In brief, equal volumes of serum and 100 TCID_50_ of vCSFV-GPE^−^/HiBiT [31] were mixed well and incubated at 37 °C for 1 h. The mixture and SK-L cell suspension were then incubated in 96-well plates at 37 °C and 5% CO_2_ for 96 h. Neutralizing antibody titers were then determined by the luciferase assay using the Nano-Glo HiBiT Lytic Detection System (Promega, Madison, WI, USA) and POWERSCAN^®^4 (Agilent Technologies International Japan Ltd., Tokyo, Japan).

### 2.10. Isolation of Porcine Peripheral Blood Mononuclear Cells (PBMCs)

Whole blood was collected in Venoject II VP-CA050K70 (Terumo) from all pigs at 7 dpv. PBMCs were prepared from whole blood by density gradient centrifugation using Ficoll-Paque PLUS (Cytiva, Marlborough, MA, USA). Cells were finally resuspended in RPMI 1640 medium supplemented with 10% fetal bovine serum (Japan Bio Serum Co., Ltd., Hiroshima, Japan) and antibiotics (Meiji Seika Pharma Co., Ltd., Tokyo, Japan).

### 2.11. In Vitro Stimulation Assay of PBMCs for the Detection of CSFV-Specific Interferon-γ (IFN-γ)-Secreting Cells by Enzyme-Linked Immunosorbent Assay (ELISA)

The in vitro stimulation assay of PBMCs was conducted following a previously described method [35]. Briefly, PBMC densities were determined and adjusted to 5 × 10^6^ cells/mL, and 200 µL of PBMCs was transferred to wells of a 96-well V-bottom plate and stimulated by 50 µL of medium containing the vGPE^−^ strain at a multiplicity of infection (MOI) of 1. A mock inoculum prepared from an uninfected SK-L cell lysate was added in an equivalent volume to the negative control samples. Cells were incubated for 72 h at 37 °C in a humidified 5% CO_2_ atmosphere, resuspended by pipetting, and centrifuged at 400× *g* for 5 min. Cell-free supernatants were removed and immediately stored at −80 °C until analysis. IFN-γ was measured in the culture supernatants using an ELISA Flex: Porcine IFN-γ (HRP) Kit according to the manufacturer’s instructions (Mabtech AB, Nacka Strand, Sweden). Absorbance at 450 nm was read using a BioTek Synergy H1 Multimode Microplate Reader (Agilent Technologies International Japan Ltd., Tokyo, Japan).

### 2.12. Statistical Analysis

Statistical analyses of the data were performed using Student’s *t*-test or one-way analysis of variance (ANOVA), followed by a post-test (Tukey’s multiple comparisons), using GraphPad Prism version 9.5.1 (San Diego, CA, USA).

### 2.13. Ethics Statement

Animal experiments were authorized by the Institutional Animal Care and Use Committee of the Faculty of Veterinary Medicine, Hokkaido University (approval number 18-0038, approved on 26 March 2018, and approval number 23-0029, approved on 23 March 2023) and performed according to the guidelines of this committee. Animals reaching the humane endpoint were euthanized by an intracardial injection of thiopental sodium (Ravonal^®^; Nipro ES Pharma Co., Ltd., Osaka, Japan) after deep sedation with isoflurane (Fujifilm Wako Pure Chemical Co., Ltd., Osaka, Japan). The experiment was conducted in animal facilities certified by the Association for Assessment and Accreditation of Laboratory Animal Care International (AAALAC International).

## 3. Results

### 3.1. Rescue of Chimeric Viruses, In Vitro Characterization, and Pathogenicity Assessment in Pigs

**Figure 1:**
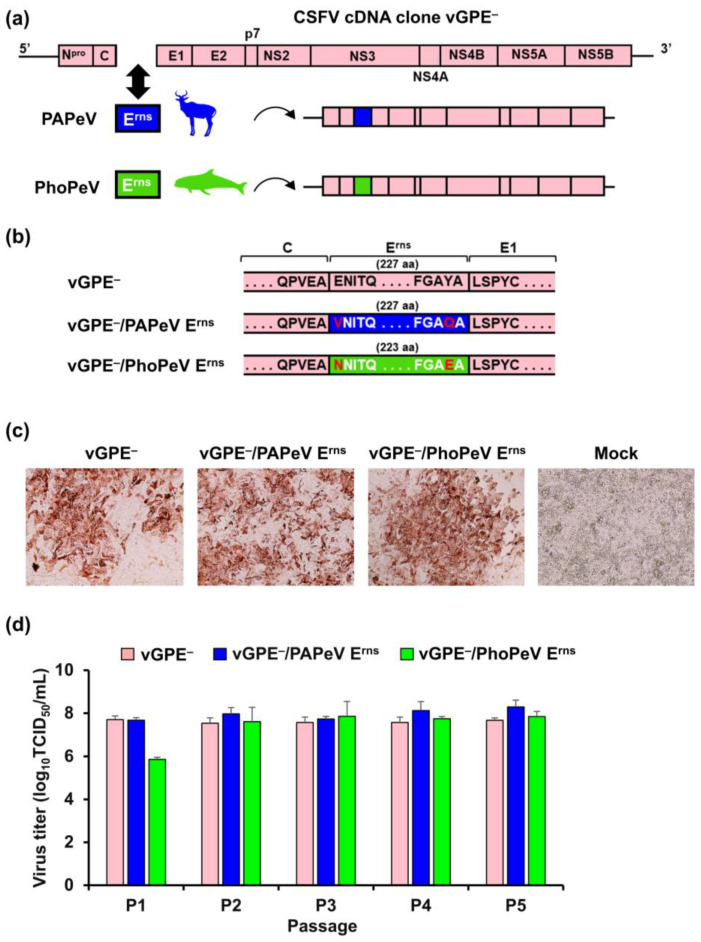
Construction and characterization of chimeric viruses in vitro. (**a**) The genomic structure of vGPE^−^ and the design of chimeric viruses, vGPE^−^/PAPeV E^rns^ and vGPE^−^/PhoPeV E^rns^ contain the E^rns^ coding sequences of PAPeV and PhoPeV, respectively. (**b**) Partial amino acid sequence alignment of the E^rns^ sequence in the viral genome of vGPE^−^/PAPeV E^rns^ or vGPE^−^/PhoPeV E^rns^ compared to the parental vGPE^−^. The different amino acids are shown in red characters at the N-terminus and C-terminus of the PAPeV E^rns^ or PhoPeV E^rns^ protein compared to those of vGPE^−^. (**c**) In vitro-transcribed RNAs derived from the viral cDNAs above were electroporated into SK-L cells. After 72 h, cells were heat-fixed and immunostained using anti-NS3 mAb 46/1. (**d**) Recombinant vGPE^−^, vGPE^−^/PAPeV E^rns^, and vGPE^−^/PhoPeV E^rns^ were passaged on SK-L cells for five rounds from the electroporated cells. After 72 h, the culture supernatants were collected and the virus titers were determined before inoculating them onto the next passage. The mean viral titers of the recombinant viruses in each passage are shown, with error bars representing standard deviations (SD; *n* = 3).

The full-length chimeric virus genomes were successfully constructed by substituting the E^rns^ sequence of the cDNA clone of CSFV vGPE^−^ with that of PAPeV or PhoPeV (Figure 1a,b). The PAPeV E^rns^ was introduced into the vGPE^−^ backbone to generate the infectious chimeric cDNA clone and the full-length linear cDNA of GPE^−^/PhoPeV E^rns^ was constructed. Subsequently, the full-length chimeric viral RNA of vGPE^−^/PAPeV E^rns^ or vGPE^−^/PhoPeV E^rns^ was transfected into SK-L cells through electroporation resulting in the expression of a viral NS3 protein as shown by IPX staining. IPX staining confirmed that these two chimeric viruses effectively infected the monolayer SK-L cell culture with vGPE^−^ used as the positive control and mock-transfected cells as the negative control (Figure 1c). Further investigations were performed in vitro to evaluate the viral growth and genetic stability of vGPE^−^/PAPeV E^rns^ and vGPE^−^/PhoPeV E^rns^ compared to the vGPE^−^ strain by conducting blind passages for five rounds in the SK-L cells. Viral growth analysis demonstrated that vGPE^−^/PAPeV E^rns^ and vGPE^−^/PhoPeV E^rns^ exhibited high and stable replication efficiency (>10^7.0^ TCID_50_) in SK-L cells to a similar extent as vGPE^−^ from the second passage (Figure 1d). In the next step, the vGPE^−^/PAPeV E^rns^ and vGPE^−^/PhoPeV E^rns^ genomes were sequenced to evaluate the genetic stability after serial passages. Both chimeric viruses displayed unchanged nucleotide sequences in the genome after five passages, indicating that vGPE^−^/PAPeV E^rns^ and vGPE^−^/PhoPeV E^rns^ maintained their efficient growth and genetic stability. In the next step, the chimeric virus vGPE^−^/PAPeV E^rns^ or vGPE^−^/PhoPeV E^rns^ was inoculated into pigs to assess the pathogenicity. As a result, no fever or clinical signs were found in all inoculated pigs (Supplementary Figure S1a). Likewise, neither typical leukopenia nor thrombocytopenia was found in pigs inoculated with vGPE^−^/PAPeV E^rns^ or vGPE^−^/PhoPeV E^rns^ during the experiment (Supplementary Figure S1b). In addition, no virus recovery was detected in blood or organ samples from all inoculated pigs (Supplementary Tables S1 and S2). Notably, neutralizing antibodies were detected in almost all pigs at the end of the experiment, except in one vGPE^−^/PhoPeV E^rns^-inoculated pig with a neutralizing antibody of less than 1 (Supplementary Table S1).

### 3.2. Infectivity and Immune Responses in Chimeric Virus-Inoculated Pigs

**Figure 2:**
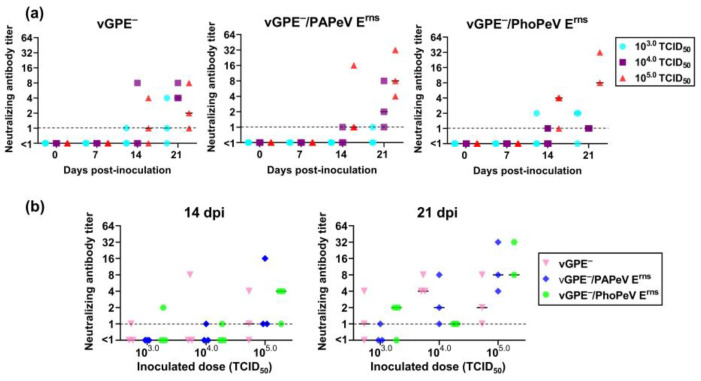
Neutralizing antibody titers in pigs inoculated with doses of 10^3.0^, 10^4.0^, and 10^5.0^ TCID_50_ at 0, 7, 14, and 21 dpi. (**a**) Neutralizing antibody titers in pigs inoculated with vGPE^−^, vGPE^−^/PAPeV E^rns^, or vGPE^−^/PhoPeV E^rns^. (**b**) Neutralizing antibody titers at 14 dpi and 21 dpi in pigs inoculated with vGPE^−^, vGPE^−^/PAPeV E^rns^, or vGPE^−^/PhoPeV E^rns^ at different doses of TCID_50_. The dot-plot graph shows individual titers with the median in each group of 10^3.0^, 10^4.0^, and 10^5.0^ TCID_50_ (*n* = 9). Dashed horizontal lines show the detection limit.

Three independent animal experiments were conducted to assess the optimal infectious dose and immune responses of chimeric virus-inoculated pigs, which would be beneficial for determining the vaccination dose in the challenge study. In each animal experiment, nine pigs were randomly divided into groups of three. Pigs were inoculated via an intramuscular route with a dose of 10^3.0^, 10^4.0^, or 10^5.0^ TCID_50_ of each virus vGPE^−^, vGPE^−^/PAPeV E^rns^, or vGPE^−^/PhoPeV E^rns^. As shown in Figure 2a, neutralizing antibodies were detected, which demonstrated a tendency to increase in most inoculated pigs from 14 dpi until the end of the experiment at 21 dpi with different immune response levels dependent on the inoculated doses, although viremia was not detected in pigs inoculated with each virus (Supplementary Table S3). In Figure 2b, either vGPE^−^/PAPeV E^rns^ or vGPE^−^/PhoPeV E^rns^ elicited CSFV-specific neutralizing antibodies comparable to the vGPE^−^ vaccine strain in pigs inoculated with 10^5.0^ TCID_50_ at 14 dpi; however, the absence of the neutralizing antibody was confirmed in several pigs that were inoculated with 10^3.0^ or 10^4.0^ TCID_50_ of each virus at this time point. Accidentally, one dead pig (#361) was found at 18 dpi without any clinical signs and no detection of virus recovery before death (Supplementary Table S3). The cause of death was unknown, as there were no gross pathological lesions or virus recovery in organ samples. After 21 days of inoculation with vGPE^−^, vGPE^−^/PAPeV E^rns^, or vGPE^−^/PhoPeV E^rns^, all pigs produced CSFV-specific neutralizing antibodies, but only those pigs inoculated with 10^4.0^ or 10^5.0^ TCID_50_ (Figure 2b). Most pigs developed immune responses at 21 dpi by inoculation with 10^3.0^ TCID_50_, except for one pig in each vaccination group of vGPE^−^ or vGPE^−^/PhoPeV E^rns^ and two vGPE^−^/PAPeV E^rns^-inoculated pigs. Our results demonstrated that vGPE^−^/PAPeV E^rns^ and vGPE^−^/PhoPeV E^rns^ induced CSFV-specific neutralizing antibody responses in pigs comparable to the parental strain vGPE^−^. Considering the vaccination dose in the subsequent challenge studies, 10^4.0^ TCID_50_ would be preferable as an optimal infectious dose for the vaccination that induced neutralizing antibodies in all pigs at the end of the experiment.

### 3.3. Efficacy of Chimeric Virus-Vaccinated Pigs against CSFV Challenge

#### 3.3.1. Vaccination Allows Solid Protection from Clinical Manifestations Following CSFV Challenge

**Figure 3:**
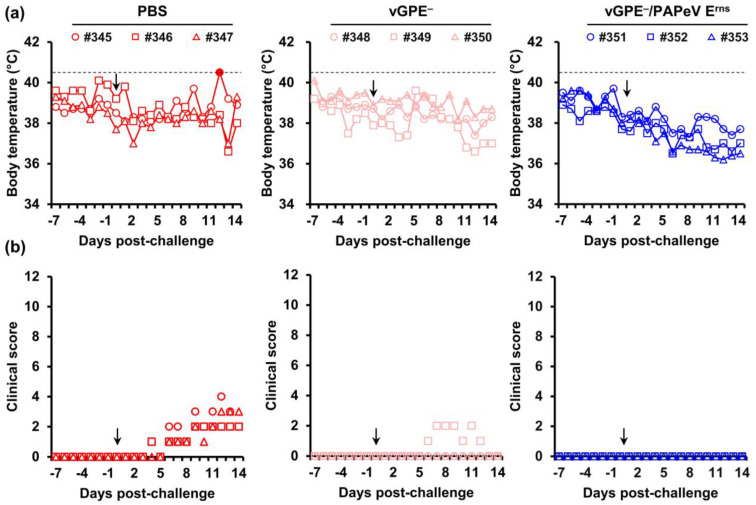
Body temperature and clinical scores of pigs in the first trial. Nine 2-week-old pigs were divided into three groups: PBS-vaccinated pigs (control) (red), vGPE^−^-vaccinated pigs (pink), and vGPE^−^/PAPeV E^rns^-vaccinated pigs (blue). (**a**) Individual body temperatures of pigs in each group. High fever was defined as a body temperature of ≥40.5 °C (dashed horizontal lines), as shown by the filled marker. (**b**) Clinical scores were monitored daily from –7 to 0 dpc until the end of the experiment. Clinical signs were scored on 10 parameters, ranging from 0 to 3 for each. The black arrow indicates the day of the virus challenge. All pigs were euthanized at 14 dpc.

**Figure 4:**
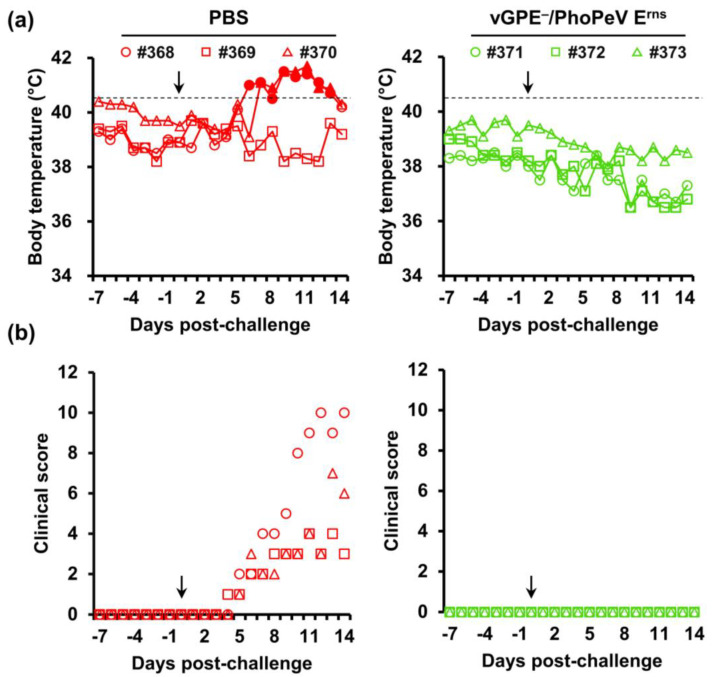
Body temperature and clinical scores of pigs in the second trial. Six 2-week-old pigs were divided into two groups: PBS-vaccinated pigs (control) (red) and vGPE^−^/PhoPeV E^rns^-vaccinated pigs (green). (**a**) Individual body temperatures of pigs in each group. High fever was defined as a body temperature of ≥40.5 °C (dashed horizontal lines), as shown by the filled markers. (**b**) Clinical scores were monitored daily from –7 to 0 dpc until the end of the experiment. Clinical signs were scored on 10 parameters, ranging from 0 to 3 for each. The black arrow indicates the day of the virus challenge. All pigs were euthanized at 14 dpc.

According to a clinical scoring system [33], there were no abnormalities in body temperature or clinical signs in all pigs until the challenge day in both trials (Figure 3 and Figure 4). In the first trial, the individual body temperatures of vaccinated or control pigs were recorded (Figure 3a). After the challenge, pigs immunized with either vGPE^−^ or vGPE^−^/PAPeV E^rns^ displayed no fever. In the control pigs, only one (#345) displayed fever (≥40.5 °C) at 12 dpc. There was also no typical sign in pigs vaccinated with vGPE^−^/PAPeV E^rns^. This phenomenon was also observed in pigs immunized with vGPE^−^, although one pig (#349) developed mild clinical signs (hesitant walking or tiredness, getting up only when forced to and lying down again) from 6 to 12 dpc but finally recovered after that until 14 dpc. In contrast, control pigs developed moderately typical CSF symptoms (diarrhea, conjunctivitis, or low appetite) from 6 dpc until the end of the experiment (Figure 3b). Similar to the first trial, no fever was observed in pigs vaccinated with vGPE^−^/PhoPeV E^rns^ after the challenge in the second trial (Figure 4a). Conversely, two control pigs (#368 and #370) had mild fever (40.3 °C) at 5 dpc which developed into acute fever (41.4 °C and 41.7 °C, respectively). Typical clinical signs were also found in control pigs in the second trial (Figure 4b), which developed mild clinical signs from 5 dpc and increased to moderate clinical signs until the end of the experiment, with the highest score of 10. Meanwhile, pigs vaccinated with vGPE^−^/PhoPeV E^rns^ showed no typical clinical signs (Figure 4b).

#### 3.3.2. Hematological Parameters in Pigs

**Figure 5:**
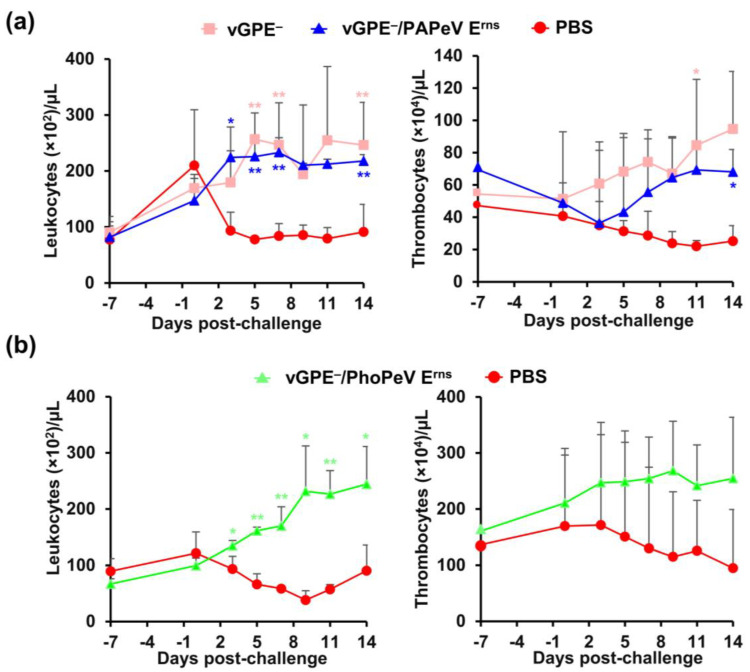
Leukocyte and thrombocyte counts of pigs in two challenge studies. (**a**) Pigs immunized with vGPE^−^ or vGPE^−^/PAPeV E^rns^ and PBS (control). (**b**) Pigs immunized with vGPE^−^/PhoPeV E^rns^ and PBS (control). Blood was collected to measure leukocyte and thrombocyte counts at each time point at −7, 0, 3, 5, 7, 9, 11, and 14 dpc. The data are shown as mean values, with error bars representing SDs. The significance of differences was calculated using one-way ANOVA, followed by the Student’s *t*-test. * *p* < 0.05; ** indicates *p* < 0.01 between the vaccinated and control groups.

Leukocyte and thrombocyte counts were utilized as an additional indicator to observe the disease manifestation of the CSFV challenge in pigs and evaluate the attenuation of chimeric viruses compared to the vGPE^−^ strain (Figure 5). Leukocyte and thrombocyte counts (Figure 5a) were dramatically decreased at 3 dpc in control pigs (93.3 ± 33.0 × 10^2^ cells/μL and 35.1 ± 14.7 × 10^4^ cells/μL, respectively) and remained at low concentrations (93.3 ± 33.0 × 10^2^ cells/μL and 35.1 ± 14.7 × 10^4^ cells/μL, respectively) until 14 dpc in the first trial. Meanwhile, in pigs immunized with either vGPE^−^ or vGPE^−^/PAPeV E^rns^ there was a tendency for an increase, but with more variation, in the leucocyte and thrombocyte counts, which were the highest in vGPE^−^-vaccinated pigs. Although slight transient thrombocytopenia was found in vGPE^−^/PAPeV E^rns^-vaccinated pigs at 3 dpc, recovery was observed later until 14 dpc. A similar tendency was found in the second trial (Figure 5b), in which vGPE^−^/PhoPeV E^rns^-vaccinated pigs showed an increase in leukocyte and thrombocyte counts from 0 dpc until the end of the experiment. In contrast, control pigs displayed a decrease in leukocyte counts and bottomed out at 9 dpc (38.3 ± 16.6 × 10^2^ cells/μL), although recovery was observed after that. Thrombocytopenia in control pigs was also observed; however, it was not significantly different from that in the vaccinated group. Taken together, the increase in leukocytes and thrombocytes in vaccinated pigs in both trials showed vaccination-induced protective immune responses, whereas control pigs maintained the status of leukopenia and thrombocytopenia.

#### 3.3.3. Early Protection in Vaccinated Pigs via IFN-γ Induction with the Absence of Neutralizing Antibody

**Figure 6:**
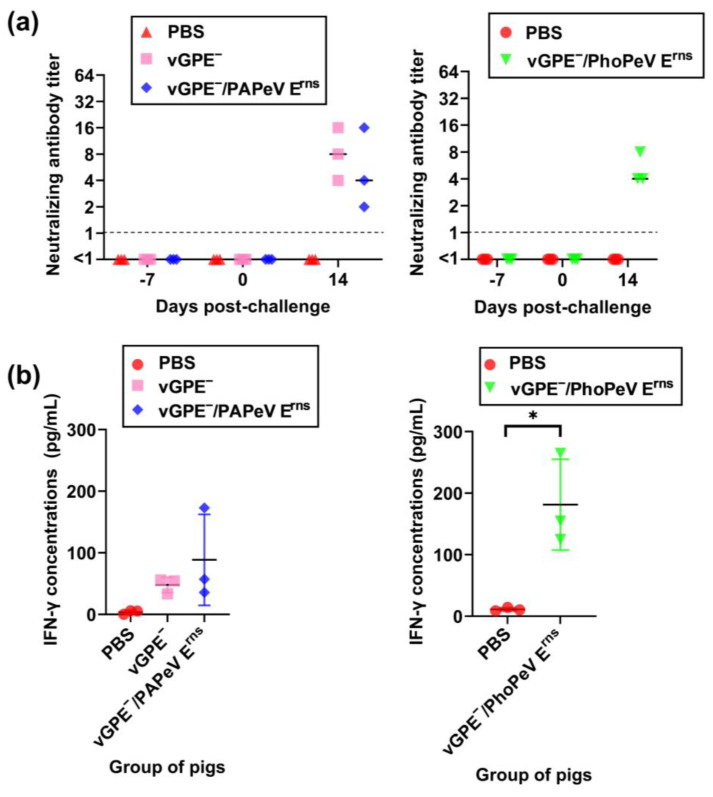
Neutralizing antibodies and cellular immune response in pigs after 7 days of vaccination. (**a**) Neutralizing antibodies in pigs vaccinated with vGPE^−^, vGPE^−^/PAPeV E^rns^, or vGPE^−^/PhoPeV E^rns^ and PBS (control). Dashed horizontal lines show the detection limit. (**b**) Detection of IFN-γ concentrations in PBMC culture supernatant by ELISA. The data are shown as mean values, with error bars representing SDs. The significance of differences was calculated using one-way ANOVA, followed by the Student’s *t*-test. * *p* < 0.05, between the vaccinated and control groups.

In both trials of the challenge study, the neutralizing antibody was under the detection limit in the control groups at 0 and 14 dpc (Figure 6a). In addition, the neutralizing antibody against vGPE^−^/PAPeV E^rns^ or vGPE^−^/PhoPeV E^rns^ was below the detection limit at 0 dpc. However, the neutralizing antibody was found in the vaccinated pigs at the end of the experiment (14 dpc), with the highest neutralizing antibody titer of 16 in vGPE^−^ or vGPE^−^/PAPeV E^rns^-vaccinated pigs, whereas pigs vaccinated with vGPE^−^/PhoPeV E^rns^ showed the highest neutralizing antibody titer of 8.

Considering the role of cellular immunity in protecting pigs against CSFV challenge at an early stage of vaccination in the absence of neutralizing antibodies, IFN-γ induction in stimulated PBMCs was evaluated in all pigs at 0 dpc. As shown in the results (Figure 6b), pigs immunized with vGPE^−^, vGPE^−^/PAPeV E^rns^, or vGPE^−^/PhoPeV E^rns^ exhibited IFN-γ production in PBMCs stimulated with vGPE^−^, whereas PBMCs from control pigs showed a deficient response to viral stimulation. In the first trial, there was no statistically significant difference between groups, although increased IFN-γ concentrations were detected in vGPE^−^ or vGPE^−^/PAPeV E^rns^-vaccinated pigs. Meanwhile, pigs immunized with vGPE^−^/PhoPeV E^rns^ exhibited IFN-γ production at a high concentration, presenting a significant difference from control pigs in the second trial.

#### 3.3.4. Protection against Systemic Infection in Vaccinated Pigs after CSFV Challenge

Before the CSFV challenge, no virus was detected in pigs immunized with vGPE^−^, vGPE^−^/PAPeV E^rns^, or vGPE^−^/PhoPeV E^rns^ and control (Table 1). Notably, the absence of viral replication in the blood (Table 1) and organ (Table 2) samples in both vGPE^−^/PAPeV E^rns^-vaccinated and vGPE^−^/PhoPeV E^rns^-vaccinated pigs was evidenced by viral titration. A transient viremia was identified in one vGPE^−^-vaccinated pig (#349) from 3 to 11 dpc (Table 1). At 14 dpc, virus recovery was confirmed in the tonsils and kidneys despite no viremia being detected in this pig (Table 2). However, the viral titer was under the detection limit of 10^1.8^ TCID_50_/g.

In contrast, control pigs in both challenge trials showed increased viremia levels in blood samples starting from 3 dpc until the end of the experiment (Table 1). The virus recovery with high titers was confirmed in most control pigs. Virus isolation in organ samples was also conducted after the necropsy (Table 2). Only one pig (in the control group) showed a moderate virus titer in organ samples (10^2.0^–10^4.0^ TCID_50_) compared to high titers of virus recovery (>10^4.0^ TCID_50_) in the other two pigs in the first trial. High virus titers (≥10^4.0^ TCID_50_) in organ samples were also confirmed in all control pigs in the second trial (Table 2). Our results demonstrated that vaccination with either vGPE^−^/PAPeV E^rns^ or vGPE^−^/PhoPeV E^rns^ could protect pigs against systemic infection from CSFV challenge.

## 4. Discussion

Up to now, the design of MLVs for the CSF marker vaccine has principally focused on substituting the E^rns^ or E2 sequence [17,18,21,36,37], deletion of virulence-associated functional residues in E^rns^ combined with the substitution of the conformational epitope in E2 of CSFV [38], modifying E1 and E2 sequences or inserting the synthetic epitope on CSFV [39,40], and swapping the CSFV C-strain 5′-UTR, 3′-UTR, and partial E2 sequence with corresponding regions of BVDV [15]. Regarding the introduction of an exogenous sequence, chimeric vaccines based on the live attenuated vaccine strains offer a promising approach to taking advantage of the DIVA strategy by incorporating a specific marker into the vaccine strains [13,36]. Although a scheme for chimeric virus construction based on the genetic and antigenic distances of pestiviruses to CSFV has been developed based on the substitution of the CSFV E^rns^ glycoprotein with that of non-CSFV pestiviruses (NRPV, PAPeV, or a combination of both) to improve the DIVA strategy, which can diminish the possibility of inducing cross-reactive antibodies [21], the assessment of safe vaccines should be considered due to the use of the virulent CSFV strain as a backbone. Therefore, new chimeric viruses for CSF marker vaccine candidates were constructed in this study by substituting the full-length glycoprotein E^rns^ with that of PAPeV or PhoPeV based on the backbone of the Japanese GPE^−^ vaccine strain used in several countries and regions for decades [12,41].

In this study, an attempt to engineer GPE^−^ into a chimeric virus vaccine candidate based on the construction of distantly related genetic pestiviruses was successfully achieved. Although there were some concerns about the malfunctioning genome or poor replication of chimeric viruses possessing E^rns^ of distantly related pestiviruses reported in previous studies [21,42], the two chimeric viruses vGPE^−^/PAPeV E^rns^ and vGPE^−^/PhoPeV E^rns^ in this study showed high viral titers and genetic stability, which are comparable to the parental strain vGPE^−^. A successful introduction of PAPeV E^rns^ into the CSFV Alfort-Tübingen backbone was previously achieved with a well-replicated chimeric virus or by swapping the E^rns^ sequence of giraffe, reindeer, or PAPeV with those of BVDV (NADL strain), indicating that the CSFV backbone could bear a substitution of E^rns^ [21,42]. Interestingly, the chimeric virus containing E^rns^ of a novel PhoPeV in marine mammals [43], which is heterologous to viruses of terrestrial mammals, could be rescued and showed efficient replication in vitro in this study. As these two chimeric viruses showed efficient replication in vitro, an investigation was conducted to assess their pathogenicity in pigs. However, the virus titer was below the detection limit in pigs, indicating that their replication was attenuated in pigs. As expected, two chimeric viruses demonstrated a safety characteristic similar to the vGPE^−^ strain reported in a previous study [25].

The immunogenicity profiles of chimeric viruses were also evaluated in pigs inoculated with different doses that could be determined as optimal vaccination doses in the challenge study. Neutralizing antibodies against CSFV were detected in all inoculated pigs without the detection of viremia, except for several pigs inoculated with the lowest TCID_50_ dose. Antibodies against the structural (E2, E^rns^) and non-structural (NS3) proteins have been detected, with E2 being the most immunogenic and essential to induce the neutralizing antibody [44,45]. In line with previous studies [21,42], substituting E^rns^ in the vGPE^−^ backbone with heterologous E^rns^ of non-CSFV pestiviruses in this study may exert less effect on the induction of neutralizing antibodies. In fact, the present experimental conditions in this study could not fulfill the vaccination guidelines and long-term observation period, which would provide enough time to induce antibody responses in pigs [23]. However, an increased level of neutralizing antibodies was observed in all inoculated pigs at all different doses of TCID_50_ during the experiment, proving that both chimeric viruses exhibited comparable immunogenicity with the parental vGPE^−^ strain.

Remarkably, this study proved its efficacy against moderately virulent CSFV challenges as early as 7 days after vaccination. In detail, pigs immunized with either the chimeric virus vGPE^−^/PAPeV E^rns^ or vGPE^−^/PhoPeV E^rns^ showed early protection against clinical signs and systemic viral replication. In contrast, control pigs exhibited fever and moderate clinical signs. The difference in the clinical score was observed in control pigs in both trials, which could be explained by the less severe clinical signs caused by the CSFV vALD-A76 challenge according to the clinical scoring system. Leukocytopenia and thrombocytopenia were observed along with long-lasting viremia by the CSFV challenge strain [27,46]. In this regard, the protective capability of chimeric viruses against CSFV systemic infection in pigs is shown to elicit an early-onset protective immune response against CSF. The advantage of a vGPE^−^-based backbone, as proven in a previous study, is that it would promote a fitting backbone in developing the CSF marker vaccine [25]. It was previously shown that the live attenuated vaccine GPE^−^ has been proven to confer early protection even at 3 dpv against CSFV challenge to prevent the development of clinical signs and viral replication [47]. The protection conferred to vaccinated pigs at 7 dpv against the CSFV challenge in this study was not solely dependent on the antibody response because the neutralizing antibody titer was undetectable in all pigs on the day of the challenge. Despite the absence of an antibody response before the CSFV challenge, neutralizing antibody titers were detected at the end of the challenge study in all vaccinated pigs compared to the lack of them in control pigs. The increase in neutralizing antibodies in vaccinated pigs could be associated with clinical and virological protection. As the activation of cell-mediated immune (CMI) responses also contributed to early protection after the CSFV challenge in pigs [35,47,48], this study investigated whether the presence of CSFV-specific IFN-γ-secreting cells could be correlated with disease protection after 7 dpv. CMI response is vital in regulating the immune response and essential for providing immunity against intracellular pathogens. In addition, the level of antigen-specific IFN-γ production could be used as an indicator of CMI response [49]. Notably, IFN-γ production from in vitro stimulated PBMCs was detected at 7 dpv in vaccinated pigs, which could not be observed clearly in the control pigs. This finding was consistent with previous studies showing that IFN-γ production could be detected in 5-week-old crossbred pigs after 6 dpv with C-strain [49]. However, recent studies have indicated that the IFN-γ secretion from PBMCs was not detected in pigs at 5 dpv, and CD4^+^ and CD8^+^ T cells were detected as the cellular source of CSFV-specific IFN-γ-secreting cells that could be seen after the CSFV challenge day [35,48]. In this study, the presence of CSFV-specific IFN-γ-secreting cells seems to be correlated with early protection against systemic infection following the CSFV challenge by 7 days after vaccination. Despite IFN-γ production on the day of the challenge as shown in this study, the crucial role of CSFV-specific antibodies should not be excluded, as there was an increased level of neutralizing antibodies in vaccinated pigs but not in control pigs. IFN-γ detection is believed to be an alternative way to assess T cell response to CSFV; however, the T cell phenotypes in PBMCs, including IFN-γ-producing CD4 or CD8 T cells, were not characterized in this study. Therefore, further studies are needed to evaluate the role and the initial source of CSFV-specific IFN-γ-producing T cells after vaccination.

This study was conducted to design and evaluate chimeric viruses with the target of providing early-onset protection against CSFV challenge. As it is also essential to prepare a serum panel at different time points in a long-term period after vaccination for test validation of DIVA potential, the antibodies against E^rns^ glycoprotein throughout these experiments were still not evaluated because of the present experimental conditions. Therefore, to differentiate chimeric vaccinated pigs from those infected with CSFV challenge, an ELISA based on CSFV E^rns^, PAPeV E^rns^, and PhoPeV E^rns^ antigens and a serum panel will be prepared to serologically detect pigs vaccinated with these two chimeric viruses, which will fulfill the DIVA strategy in future work.

In conclusion, a combination of a live attenuated CSFV vaccine strain with the glycoprotein E^rns^, which is distantly related to CSFV, resulted in two chimeric viruses, vGPE^−^/PAPeV E^rns^ and vGPE^−^/PhoPeV E^rns^, capable of quick and efficient protection from systemic infection by moderately virulent CSFV challenges. Although the DIVA properties were not evaluated for these chimeric viruses, their great potential could be promoted in the joint development of marker vaccines. Future work should address the safety, efficacy, and DIVA properties of these two chimeric viruses to develop a CSF marker vaccine.

## Figures and Tables

**Table 1:** Virus recovery from blood samples in pigs vaccinated with vGPE^−^, vGPE^−^/PAPeV E^rns^, or vGPE^−^/PhoPeV E^rns^ and PBS (control).

Trial	Virus	Pig ID	Virus Recovery at dpc (log_10_ TCID_50_/mL)							
			−7	0	3	5	7	9	11	14
First	vGPE^−^/PAPeV E^rns^	#351	–	–	–	–	–	–	–	–
		#352	–	–	–	–	–	–	–	–
		#353	–	–	–	–	–	–	–	–
	vGPE^−^	#348	–	–	–	–	–	–	–	–
		#349	–	–	+	2.8	3.3	1.3	+	–
		#350	–	–	–	–	–	–	–	–
	Control	#345	–	–	1.8	3.6	4.6	6.0	6.1	6.8
		#346	–	–	1.5	2.8	3.6	3.8	3.3	2.8
		#347	–	–	1.3	3.6	4.3	6.3	5.8	6.0
Second	vGPE^−^/PhoPeV E^rns^	#371	–	–	–	–	–	–	–	–
		#372	–	–	–	–	–	–	–	–
		#373	–	–	–	–	–	–	–	–
	Control	#368	–	–	2.3	4.3	5.8	5.8	6.0	5.6
		#369	–	–	1.6	3.3	3.8	4.3	4.8	4.0
		#370	–	–	1.3	4.0	6.0	6.5	6.3	6.6

–: not isolated; +: isolated in a 6-well plate and lower than the detection limit of TCID_50_ (10^0.8^ TCID_50_/mL) in a 96-well plate.

**Table 2:** Virus recovery from organ samples in pigs vaccinated with vGPE^−^, vGPE^−^/PAPeV E^rns^, or vGPE^−^/PhoPeV E^rns^ and PBS (control).

Trial	Virus	Pig ID	Virus Recovery (log_10_ TCID_50_/g)						
			Tonsil	Brain	Spleen	Kidney	Adrenal Grand	Mesenteric Lymph Node	Colon
First	vGPE^−^/PAPeV E^rns^	#351	–	–	–	–	–	–	–
		#352	–	–	–	–	–	–	–
		#353	–	–	–	–	–	–	–
	vGPE^−^	#348	–	–	–	–	–	–	–
		#349	+	–	–	+	–	–	–
		#350	–	–	–	–	–	–	–
	Control	#345	6.0	4.8	6.1	5.3	5.0	6.6	6.1
		#346	3.3	2.7	2.7	3.8	2.6	2.3	2.3
		#347	6.1	4.3	7.3	5.5	5.6	5.8	5.5
Second	vGPE^−^/PhoPeV E^rns^	#371	–	–	–	–	–	–	–
		#372	–	–	–	–	–	–	–
		#373	–	–	–	–	–	–	–
	Control	#368	5.0	4.0	6.0	5.8	5.8	5.0	5.0
		#369	4.6	4.0	4.8	5.0	4.8	5.0	4.8
		#370	6.3	4.3	6.6	6.8	6.8	5.8	5.0

–: not isolated, +: isolated in a 6-well plate and lower than the detection limit of TCID_50_ (10^1.8^ TCID_50_/g) in a 96-well plate.

## Data Availability

Not applicable.

## Supplementary Materials

The following supporting information can be downloaded at: https://www.mdpi.com/article/10.3390/v15071587/s1, Figure S1: Body temperature, clinical score, leukocyte and thrombocyte counts of pigs inoculated with the vGPE^−^/PAP E^rns^ or vGPE^−^/PAP E^rns^; Table S1: Virus recovery from blood samples and neutralizing antibody in pigs inoculated with vGPE^−^/PAPeV E^rns^ or vGPE^−^/PhoPeV E^rns^; Table S2: Virus recovery from organ samples in pigs inoculated with vGPE^−^/PAPeV E^rns^ or vGPE^−^/PhoPeV E^rns^; Table S3: Virus recovery from blood samples in pigs inoculated with vGPE^−^, vGPE^−^/PAPeV E^rns^, or vGPE^−^/PhoPeV E^rns^ at different doses of TCID_50_.
