# Supplementary material for: Generation and Efficacy of Two Chimeric Viruses Derived from GPE− Vaccine Strain as Classical Swine Fever Vaccine Candidates
Source: Viruses. 2023 Jul 20;15(7):1587. doi: 10.3390/v15071587 (PMC10384557; doi:10.3390/v15071587)
Supplement: Supplementary file 1 [file viruses-15-01587-s001.zip › Figure S1.pdf]

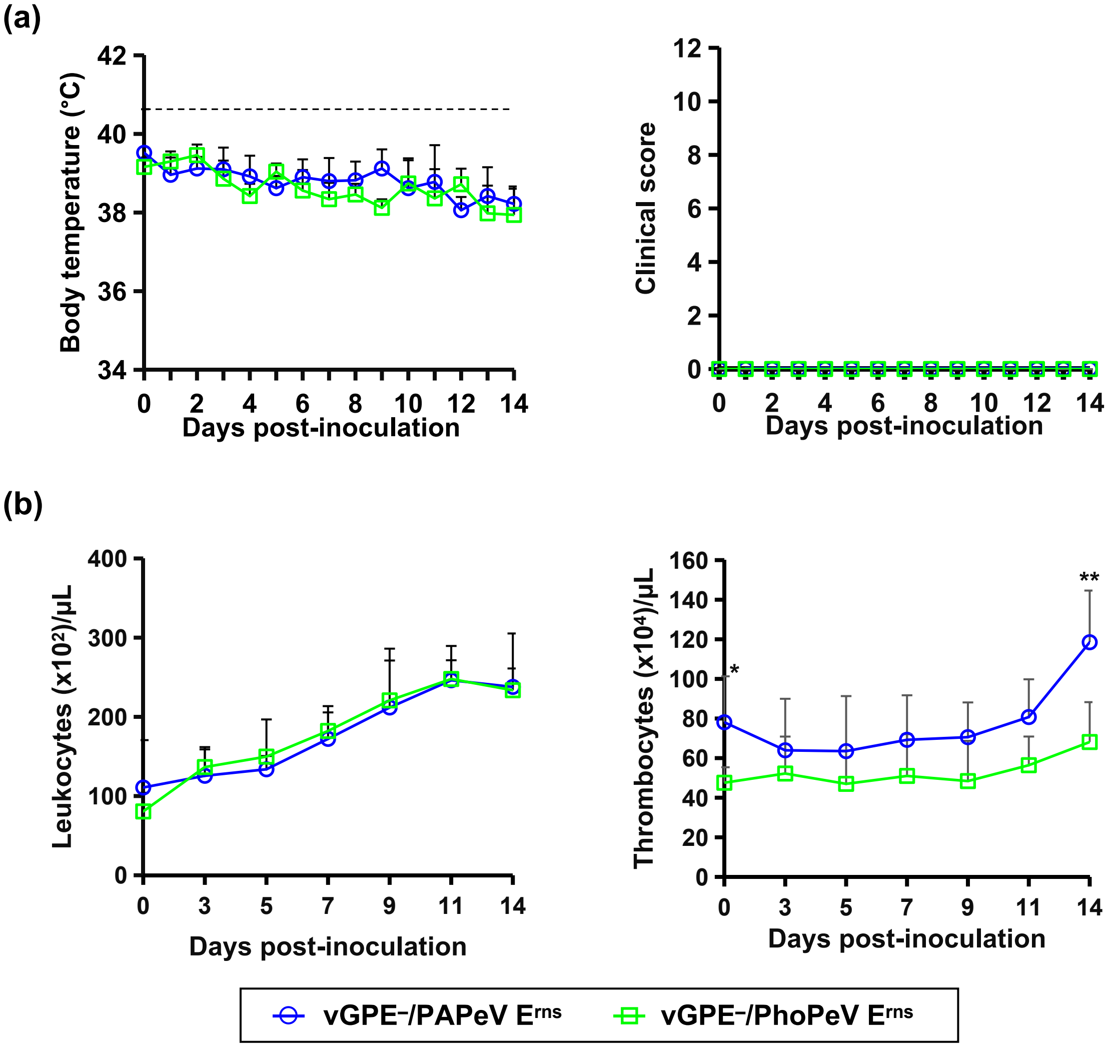

Figure S1. Body temperature, clinical score, leukocyte and thrombocyte counts of pigs inoculated with the vGPE⁻/PAP Eʳⁿˢ or vGPE⁻/PAP Eʳⁿˢ. Groups of 5 pigs were inoculated with each virus, and blood was collected on 0, 3, 5, 7, 9, 11, and 14 dpi. **(a)** The body temperature and clinical score were daily monitored. High fever was defined as a body temperature of ≥ 40.5 °C (dashed horizontal lines). **(b)**The leukocyte and thrombocyte counts were measured at each time point. All objects are shown as mean values, with error bars representing the standard deviations. The significance of differences was calculated by the Student's t-test. *$p < 0.05$; ** indicates $p < 0.01$ between the vaccinated groups.
