# Supplementary material for: Generation and Efficacy of Two Chimeric Viruses Derived from GPE− Vaccine Strain as Classical Swine Fever Vaccine Candidates
Source: Viruses. 2023 Jul 20;15(7):1587. doi: 10.3390/v15071587 (PMC10384557; doi:10.3390/v15071587)
Supplement: Supplementary file 1 [file viruses-15-01587-s001.zip › Supplementary Tables.pdf]

Table S1. Virus recovery from blood samples and neutralizing antibody in pigs inoculated with vGPE⁻/PAPeV E^{rns} or vGPE⁻/PhoPeV E^{rns}

Virus	Pig ID	Virus recovery at dpi (log_{10} $TCID_{50}$/mL)							Neutralizing antibody titer at dpi	
		0	3	5	7	9	11	14	0	14
vGPE⁻/PAPeV E^{rns}	#293	–	–	–	–	–	–	–	<1	2
	#294	–	–	–	–	–	–	–	<1	8
	#295	–	–	–	–	–	–	–	<1	4
	#296	–	–	–	–	–	–	–	<1	2
	#297	–	–	–	–	–	–	–	<1	8
vGPE⁻/PhoPeV E^{rns}	#374	–	–	–	–	–	–	–	<1	1
	#375	–	–	–	–	–	–	–	<1	2
	#376	–	–	–	–	–	–	–	<1	2
	#377	–	–	–	–	–	–	–	<1	2
	#378	–	–	–	–	–	–	–	<1	<1

–: not isolated.

Table S2. Virus recovery from organ samples in pigs inoculated with vGPE^{-}/PAPeV E^{rns} or vGPE^{-}/PhoPeV E^{rns}

Virus	Pig ID	Virus recovery ($\log_{10}$ $TCID_{50}$/g)						
		Tonsil	Brain	Spleen	Kidney	Adrenal grand	Mesenteric lymph node	Colon
vGPE^{-}/PAPeV E^{rns}	#293	–	–	–	–	–	–	–
	#294	–	–	–	–	–	–	–
	#295	–	–	–	–	–	–	–
	#296	–	–	–	–	–	–	–
	#297	–	–	–	–	–	–	–
vGPE^{-}/PhoPeV E^{rns}	#374	–	–	–	–	–	–	–
	#375	–	–	–	–	–	–	–
	#376	–	–	–	–	–	–	–
	#377	–	–	–	–	–	–	–
	#378	–	–	–	–	–	–	–

–: not isolated.

Table S3. Virus recovery from blood samples in pigs inoculated with vGPE⁻, vGPE⁻/PAPeV E^{rns}, or vGPE⁻/PhoPeV E^{rns} at different doses of $TCID_{50}$

Virus	$TCID_{50}$	Pig ID	Virus recovery at dpi (log_{10} $TCID_{50}$/mL)			
			0	7	14	21
vGPE⁻/PAPeV E^{rns}	$10^{3.0}$	#312	–	–	–	–
		#313	–	–	–	–
		#314	–	–	–	–
	$10^{4.0}$	#315	–	–	–	–
		#316	–	–	–	–
		#317	–	–	–	–
	$10^{5.0}$	#318	–	–	–	–
		#319	–	–	–	–
		#320	–	–	–	–
vGPE⁻/PhoPeV E^{rns}	$10^{3.0}$	#354	–	–	–	–
		#355	–	–	–	–
		#356	–	–	–	–
	$10^{4.0}$	#357	–	–	–	–
		#358	–	–	–	–
		#359	–	–	–	–
	$10^{5.0}$	#360	–	–	–	–
		#361	–	–	–	NC
		#362	–	–	–	–
vGPE⁻	$10^{3.0}$	#303	–	–	–	–
		#304	–	–	–	–
		#305	–	–	–	–
	$10^{4.0}$	#306	–	–	–	–
		#307	–	–	–	–
		#308	–	–	–	–
	$10^{5.0}$	#309	–	–	–	–
		#310	–	–	–	–
		#311	–	–	–	–

–: not isolated, NC: no sample collection due to the pig being dead on 18 dpi.
